# Supplementary material for: High residual carriage of vaccine-serotype Streptococcus pneumoniae after introduction of pneumococcal conjugate vaccine in Malawi
Source: Nat Commun. 2020 May 6;11:2222. doi: 10.1038/s41467-020-15786-9 (PMC7203201; doi:10.1038/s41467-020-15786-9)
Supplement: Supplementary file 1 — Supplementary Information [file 41467_2020_15786_MOESM1_ESM.pdf]

Supplementary Information

Residual carriage of vaccine-serotype Streptococcus pneumoniae after introduction of a pneumococcal conjugate vaccine in Malawi

Swarthout, T. D. et al.

Supplementary Table 1. Regional differences in vaccine formulations and schedules

Country	WHO region	World Bank income group	Year PCV introduced	Current formulation	Current dosing schedule	Current timing	Initial formulation (if different than current)
Central African Republic	AFRO	Low income	2011	PCV13	3+0	6, 10, 14 weeks	--
Ethiopia	AFRO	Low income	2011	PCV10	3+0	6, 10, 14 weeks	--
Gambia	AFRO	Low income	2009	PCV13	3+0	2, 3, 4 months	PCV7
Kenya	AFRO	Lower middle income	2011	PCV10	3+0	6, 10, 14 weeks	--
Lesotho	AFRO	Lower middle income	2015	PCV13	3+0	6, 10, 14 weeks	--
Madagascar	AFRO	Low income	2012	PCV10	3+0	6, 10, 14 weeks	--
Mozambique	AFRO	Low income	2013	PCV13	3+0	6, 10, 14 weeks	PCV10
Namibia	AFRO	Upper middle income	2014	PCV13	3+0	6, 10, 14 weeks	--
Rwanda	AFRO	Low income	2009	PCV13	3+0	6, 10, 14 weeks	PCV7
South Africa	AFRO	Upper middle income	2009	PCV13	2+1	6, 14 weeks & 9 months	PCV7
Tanzania	AFRO	Low income	2012	PCV13	3+0	6, 10, 14 weeks	--
Uganda	AFRO	Low income	2013	PCV10	3+0	6, 10, 14 weeks	--
Zambia	AFRO	Lower middle income	2013	PCV10	3+0	6, 10, 14 weeks	--
Zimbabwe	AFRO	Lower middle income	2012	PCV13	3+0	6, 10, 14 weeks	--
Canada*	AMRO	High income	2002	PCV13	2+1	2, 4, 6 months	PCV7
					3+1	2, 4, 6, & 12-18 months	--
Haiti	AMRO	Low income	2018	PCV13	3+0	6, 10, 14 weeks	--
Mexico	AMRO	Upper middle income	2008	PCV13	2+1	2, 4, & 12 months	PCV7
Nicaragua	AMRO	Lower middle income	2010	PCV13	3+0	2, 4, 6 months	--
United States	AMRO	High income	2000	PCV13	3+1	2, 4, 6 & >12 months	PCV7
Kuwait	EMRO	High income	2007	PCV13	3+1	2, 4, 6 & 18 months	PCV7
Morocco	EMRO	Lower middle income	2010	PCV10	2+1	2, 4 & 12 months	--
Saudi Arabia	EMRO	High income	2009	PCV13	3+1	2, 4, 6 & 12 months	PCV7
Denmark	EURO	High income	2007	PCV13	2+1	3, 5 & 12 months	--
France	EURO	High income	2006	PCV13	2+1	2, 4 & 11 months	PCV7
Germany	EURO	High income	2006	PCV13	2+1	2, 4 & 11-14 months	PCV7
Ireland	EURO	High income	2008	PCV13	2+1	2, 6 & 12 months	PCV7
Israel	EURO	High income	2009	PCV13	2+1	2, 4 & 12 months	PCV7
Italy	EURO	High income	2005		2+1	3, 5-6 & 11-13 months	PCV7
Netherlands	EURO	High income	2006	PCV10	2+1 (3+1)	2, 4 & 11 months	PCV7
Portugal	EURO	High income	2015	PCV13	2+1	2, 4 & 12 months	
Russian Federation	EURO	Upper middle income	2014	PCV13	2+1	2, 4.5 & 15 months	PCV10
Spain	EURO	High income	2001	PCV13	2+1 (3+1)	2, 4 & 12 months	PCV7
Switzerland	EURO	High income	2006	PCV13	2+1	2, 4 & 12 months	PCV7
United Kingdim	EURO	High income	2006	PCV13	2+1	2, 4 & 13 months	PCV7
Bangladesh	SEARO	Lower middle income	2015	PCV10	3+0	6, 10, 14 weeks	--
India	SEARO	Lower middle income	2017	PCV13	2+1	6, 14 weeks & 9 months	--
Indonesia	SEARO	Lower middle income	2018	PCV13	2+1	2, 3 & 12 months	--
Myanmar	SEARO	Lower middle income	2016	PCV13	3+0	2, 4, 6 months	PCV10
Nepal	SEARO	Low income	2015	PCV10	2+1	6, 10 weeks & 9 months	--
Australia	WPRO	High income	2005	PCV13	2+1	2, 4, & 12 months	PCV7
Cambodia	WPRO	Lower middle income	2015	PCV13	3+0	6, 10, 14 weeks	--
Fiji	WPRO	Upper middle income	2012	PCV10	3+0	6, 10, 14 weeks	--
Japan	WPRO	High income	2011	PCV13	3+1	2, 3, 4 months & 1 year	PCV7
New Zealand	WPRO	High income	2008	PCV13	3+1	6 weeks, 3, 5, & 15 months	PCV7

Data collected from: http://www.view-hub.org/

*Canada: According to Jan 2016 schedule, Northwest Territories and Nunavut province use a 3+1 schedule (2, 4, 6, 18 months); all the other provinces use a 2+1 schedule (either 2, 4, 12 months or 2, 4, 18 months).

Supplementary Table 2. Regional VT carriage prevalence, pre- and post-PCV introduction

Country	Region	Carriage Prevalence Pre-Vaccine	Carriage Prevalence Post-Vaccine	Percent Reduction	Time (years) since PCV introduction	Age	PCV	Ref
Fiji	WPRO	22.3	5.8	74.0	3	12-23 m	PCV10	[1]
Australia	WPRO	23.7	12.0	49.4	1.5	< 5y	PCV13	[2]
Mozambique	AFRO	35.1	20.9	40.5	2	24-59 m	PCV10	[3]
Kenya	AFRO	33.8	13	61.5	2	< 5y	PCV10	[4]
Kenya	AFRO	"	8.8	73.9	5	"	"	"
Malawi	AFRO	28.2	17.9	36.6	4.5	1-4 y	PCV13	[5]
USA	AMRO	55.4	10.9	80.3	2	<5 y	PCV7	[6]
USA	AMRO	"	4.8	91.4	3	"	"	"
Netherlands	EURO	38	8	80.1	3	11 m	PCV7	[7]
Netherlands	EURO	36	4	88.1	3	2 y	PCV7	"
UK	EURO	31.9	4.2	86.4	7	<5 y	PCV7	[8]
UK	EURO	"	0.4	98.9	11	"	"	"
UK	EURO	39.9	1.0	97.5	7	<5 y	PCV13	[9]

“Indicates that the information for this cell is the same as the information in the cell directly above.

Supplementary Table 3. PCV-vaccinated study groups: Pneumococcal carriage prevalence, stratified by survey

18 weeks – 1 year old (PCV-vaccinated)	Survey 1[§]	Survey 2[§]	Survey 3[§]	Survey 4 (n=153)	Survey 5 (n=147)	Survey 6 (n=139)	Survey 7 (n=127)	Total (n=566)
Total carriage								
% (n)	--	--	--	77·1 (118)	82·3 (121)	79·1 (110)	81·1 (103)	79·9 (452)
95% CI				69·6, 83·5	75·2, 88·1	71·4, 85·6	73·2, 87·5	76·3, 83·1
VT								
	--	--	--	19·0 (29)	17·7 (26)	16·5 (23)	15·0 (19)	17·1 (97)
				13·5, 26·0	12·3, 24·8	11·2, 23·7	9·7, 22·3	14·2, 20·5
NVT								
	--	--	--	58·1 (89)	64·6 (95)	62·6 (87)	66·1 (84)	62·7 (355)
				50·2, 65·8	56·6, 72·9	54·2, 70·3	57·4, 73·9	58·6, 66·6

2 years old (PCV-vaccinated)	Survey 1[§]	Survey 2[§]	Survey 3[§]	Survey 4 (n=124)	Survey 5 (n=114)	Survey 6 (n=135)	Survey 7 (n=126)	Total (n=499)
Total carriage								
% (n)	--	--	--	78·3 (97)	83·3 (95)	71·8 (97)	75·4 (95)	76.9 (384)
95% CI				69·9, 85·1	75·2, 89.6	63·5, 79·2	66·9, 82·6	73·0, 80·6
VT								
	--	--	--	21·8 (27)	18·4 (21)	18·5 (25)	15·1 (19)	18·4 (92)
				15·3, 30·0	12·3, 26·7	12·8, 26·0	9·8, 22·5	15·3, 22·1
NVT								
	--	--	--	56·5 (70)	64·9 (74)	53·3 (72)	60·3 (76)	58·5 (292)
				47·6, 64·9	55·7, 73·2	44·9, 61·6	51·5, 68·5	54·1, 62·8

3–7 years old (PCV-vaccinated)	Survey 1 (n=286)	Survey 2 (n=303)	Survey 3 (n=361)	Survey 4 (n=380)	Survey 5 (n=382)	Survey 6 (n=475)	Survey 7 (n=378)	Total (n=2565)
Total carriage								
% (n)	84·2 (241)	76·0 (230)	78·1 (282)	66·8 (254)	80·4 (307)	61·7 (293)	78·1 (295)	74·2 (1902)
95% CI	79·5, 88·3	70·7, 80.6	73·5, 82·3	61·9, 71·6	76·0, 84·2	57·4, 66·1	73·5, 82·1	72·4, 75·8
VT								
	19·9 (57)	20·5 (62)	20·8 (75)	17·6 (67)	19·4 (74)	13·3 (63)	16·7 (63)	18·0 (461)
	15·7, 25·0	16·3, 25·4	16·9, 25·3	14·1, 21·8	15·7, 23·7	10·5, 16·6	13·2, 20·8	16·5, 19·5
NVT								
	64·3 (184)	55·5 (168)	57·3 (207)	49·2 (187)	61·0 (233)	48·4 (230)	61·4 (232)	56·2 (1441)
	58·6, 69·7	49·8, 61·0	52·2, 62·4	44·2, 54·2	56·0, 65·8	43·9, 52·9	56·4, 66·2	54·2, 58·1

[§] There was no recruitment for these age groups during these surveys. CI=confidence interval. VT=vaccine serotype. NVT=non-vaccine serotype.

Supplementary Table 4. PCV-unvaccinated study groups: Pneumococcal carriage prevalence, stratified by survey

4-8 weeks old (PCV-unvaccinated)	Survey 1§	Survey 2§	Survey 3§	Survey 4§	Survey 5 (n=121)	Survey 6 (n=133)	Survey 7 (n=92)	Total (n=346)
Total carriage								
% (n)	-	-	-	-	42·9 (52)	30·8 (41)	57·6 (53)	42·2 (146)
95% CI					34.0, 52.3	23.1, 39.4	46.9, 67.9	36.9, 47.6
VT	-	-	-	-	10·7 (13)	6·0 (8)	8·7 (8)	8·4 (29)
					6·3, 17·7	3·0, 11·6	4·4, 16·5	5·7, 11·8
NVT	-	-	-	-	32·2 (39)	24·8 (33)	48·9 (45)	33·8 (117)
					24·5, 41·1	18·2, 32·9	38·8, 59·1	28·8, 39·1

3–10 years old (PCV-unvaccinated)	Survey 1 (n=255)	Survey 2 (n=231)	Survey 3 (n=242)	Survey 4 (n=198)	Survey 5 (n=106)	Survey 6 (n=173)	Survey 7 (n=197)	Total (n=1402)
Total carriage								
% (n)	68·3 (174)	62·4 (144)	58·7 (142)	37·8 (75)	58·5 (62)	48·6 (84)	57·4 (113)	56·7 (794)
95% CI	62·1, 73·9	55·7, 68·6	52·2, 64·9	31·1, 45·0	48·5, 68·0	40·9, 56·3	50·1, 64·4	53·9, 59·2
VT	27·5 (70)	21·7 (50)	19·4 (47)	14·1 (28)	10·4 (11)	11·0 (19)	15·2 (30)	18·2 (255)
	22·3, 33·3	16·7, 27·4	14·9, 24·9	9·9, 19·7	5·8, 17·8	7·1, 16·6	10·8, 21·0	16·2, 20·3
NVT	40·8 (104)	40·7 (94)	39·3 (95)	23·7 (47)	48·1 (51)	37·6 (65)	42·1 (83)	38·5 (539)
	34·9, 46·9	34·5, 47·2	33·2, 45·6	18·3, 30·2	38·7, 57·6	30·6, 45·0	35·4, 49·2	35·9, 41·0

18-40 years old (PCV-unvaccinated)	Survey 1 (n=198)	Survey 2 (n=201)	Survey 3 (n=279)	Survey 4 (n=308)	Survey 5 (n=305)	Survey 6 (n=277)	Survey 7 (n=202)	Total (n=1770)
Total carriage								
% (n)					38.4 ()	32.5 (90)	38.6 (78)	40.4 (714)
95% CI								
VT	15·2 (30)	14·4 (29)	14·0 (39)	14·3 (44)	10·5 (32)	9·0 (25)	8·9 (18)	12·3 (217)
	10·8, 20·9	10·2, 20·0	10·4, 18·6	10·8, 18·9	7·5, 14·5	6·2, 13·0	5·7, 13·7	10·8, 13·9
NVT	24·2 (48)	32·8 (66)	30·5 (85)	28·6 (88)	27·9 (85)	23·5 (65)	29·7 (60)	28·1 (497)
	18·8, 30·7	26·7, 39·6	25·3, 36·1	23·8, 33·9	23·1, 33·2	18·8, 28·8	23·8, 36·4	26·0, 30·2

§ There was no recruitment for these age groups during these surveys. CI=confidence interval. VT=vaccine serotype. NVT=non-vaccine serotype.

Supplementary Table 5. Population-based pneumococcal carriage prevalence studies, Malawi

Age	Carriage	Karonga (rural), Malawi[1] 2009-2011 Pre PCV intro.	Karonga (rural), Malawi[1] 2014 Post PCV intro.		Blantyre (urban), Malawi 2015-2019 Post PCV intro.
6 weeks old PCV-unvaccinated[2]	Spn	38·6	43·8		57·3
	VT	11·4	13·0		8·4
	NVT	27·1	30·8		48·9
	Total N	70	146		346
18 weeks old PCV-vaccinated[3]	Spn	74·7	50·0		81·8
	VT	45·1	9·1		16·8
	NVT	29·6	40·9		65·0
	Total N	71	44		203
			Unvacc'd	Vacc'd	Vacc'd
1-4 years old PCV-unvaccinated and vaccinated	Spn	59·4	63·9	70·0	77·2
	VT	28·2	22·9	16·5	18·7
	NVT	31·2	41·0	53·4	58·5
	Total N	330	83	103	2958
			Unvacc'd[4]	Vacc'd	
18 weeks-4years (<5y) PCV-unvaccinated and vaccinated	Spn	62·1	63·9	63·9	77·5
	VT	31·2	22·9	14·3	18·6
	NVT	30·9	41·0	49·7	58·9
	Total N	401	83	147	3161
5-15 years PCV-unvaccinated[5]	Spn	49·5	37·1		53·8
	VT	21·2	7·9		16·3
	NVT	28·3	29·2		37·5
	Total N	325	89		1748

[1] Heinsbroek *et al.* Ref [5]. [2] Blantyre study data includes ages 4 – 8 weeks of age. [3] Blantyre study data includes ages 18 weeks – 12 months of age. [4]No children <1yrs unvaccinated, therefore same results as among children 1-4 years old. [5]Blantyre study data among PCV-unvaccinated is limited to ages 3 – 10 years. PCV=pneumococcal conjugate vaccine. intro.=introduction. Spn=*Streptococcus pneumoniae*. VT=vaccine serotype. NVT=non-vaccine serotype. Vacc'd=PCV-vaccinated. Unvacc'd=PCV-unvaccinated.

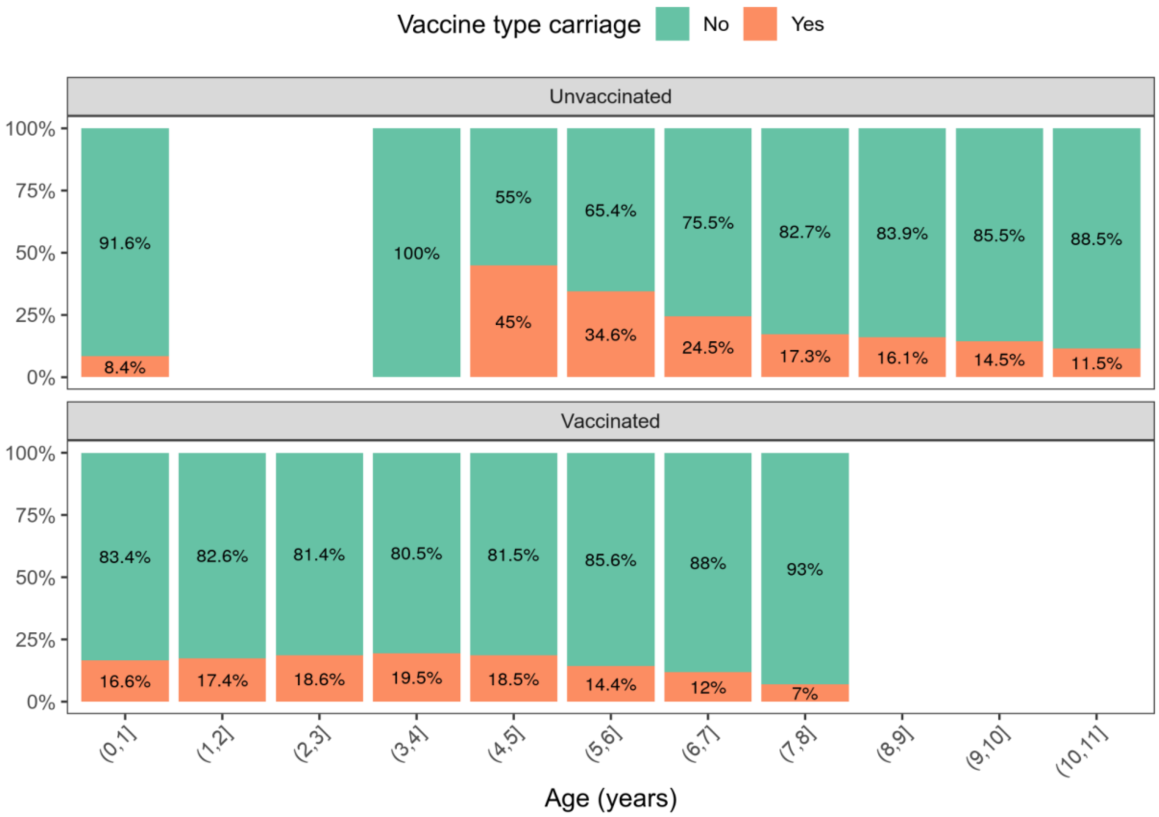

Supplementary Figure 1. VT and NVT carriage among PCV-vaccinated and PCV-unvaccinated children. Proportions reported in the lower (orange) section of each bar represent the prevalence of VT carriage in each age group. Proportions reported in the upper (green) section of each bar represent the prevalence of no VT carriage (NVT + no carriage). The (0, 1] bar among unvaccinated children includes only children 4-8 weeks of age. The (A, B] notation used for each age group includes all children whose age was greater than A and less than or equal to B (A < age ≤ B). When modelling the entire range of data (i.e. not censoring at 3·6 years) with an exponential decay curve the estimated decay parameter for vaccinated was not significantly different from zero (i.e. an almost flat curve was the resulting fit). Among PCV-vaccinated children there is an observed increase in pneumococcal carriage until the (3,4] year age range and only then does VT carriage prevalence start to decrease. Our conjecture is that the overall trend in pneumococcal carriage for vaccinated children starting from birth is more complex than an exponential decay curve.

Supplementary Table 6. Denominator (N) for each age bar in Supplementary Figure 1

	(0, 1]	(1, 2]	(2, 3]	(3, 4]	(4, 5]	(5, 6]	(6, 7]	(7, 8]	(8, 9]	(9, 10]	(10, 11]
Unvaccinated, N	346	--	--	2	16	136	150	243	302	272	281
VacciANTed, N	203	363	499	1,105	991	309	117	43	--	--	--

For cells showing ‘--‘, there was no recruitment for these age groups.

Supplementary Table 7. Proportion VT carriage attributed to individual VT (PCV-vaccinated)

Vaccine serotype	Survey-1 % (n)	Survey-2 % (n)	Survey-3 % (n)	Survey-4 % (n)	Survey-5 % (n)	Survey-6 % (n)	Survey-7 % (n)	Total % (n)
Children 18 weeks to 1 year, PCV-vaccinated §								
1	--	--	--	0	0	(4·4) 1	0	1·0 (1)
3	--	--	--	6·9 (2)	7·7 (2)	8·7 (2)	21·0 (4)	10·3 (10)
4	--	--	--	0	0	0	5·3 (1)	1·0 (1)
5	--	--	--	0	0	0	0	0
6A	--	--	--	20·7 (6)	15·4 (4)	21·7 (5)	15·8 (3)	18·6 (18)
6B	--	--	--	13·8 (4)	3·9 (1)	0	0	5·2 (5)
7F	--	--	--	0	3·9 (1)	0	0	1·0 (1)
9V	--	--	--	3·5 (1)	7·7 (2)	4·4 (1)	5·3 (`)	5·2 (5)
14	--	--	--	13·8 (4)	15·4 (4)	34·8 (8)	26·3 (5)	21·7 (21)
18C	--	--	--	0	0	0	5·3 (1)	1·0 (1)
19A	--	--	--	13·8 (4)	3·9 (1)	8·7 (2)	0	7,2 (7)
19F	--	--	--	13·8 (4)	23·1 (6)	17·4 (4)	10·5 (2)	16·5 (16)
23F	--	--	--	13·8 (4)	19·2 (5)	0	10·5 (2)	11·3 (11)
Total	--	--	--	100 (29)	100 (26)	100 (23)	100 (19)	100 (97)
Children 2 years, PCV-vaccinated §								
1	--	--	--	0	(2)	0	0	2·2 (2)
3	--	--	--	18·5 (5)	14·3 (3)	16·0 (4)	10·5 (2)	15·2 (14)
4	--	--	--	0	0	0	10·5 (2)	2·2 (2)
5	--	--	--	()	()	()	()	()
6A	--	--	--	22·2 (6)	9·5 (2)	8·0 (2)	15·8 (3)	14·1 (13)
6B	--	--	--	()	()	()	()	()
7F	--	--	--	()	()	()	()	()
9V	--	--	--	7·4 (2)	9·5 (2)	0	5·3 (1)	5·4 (5)
14	--	--	--	11·1 (3)	14·3 (3)	28·0 (7)	21·1 (4)	18·5 (17)
18C	--	--	--	3·7 (1)	4·8 (1)	4·0 (1)	0	3·3 (3)
19A	--	--	--	7·4 (2)	0	4·0 (1)	5·3 (1)	4·4 (4)
19F	--	--	--	25·9 (7)	14·3 (3)	28·0 (7)	26·3 (5)	23·9 (22)
23F	--	--	--	3·7 (1)	23·8 (5)	12·0 (3)	5·3 (1)	10·9 (10)
Total	--	--	--	(100) 27	(100) 21	(100) 25	(100) 19	(100) 92
Children 3-7 years, PCV-vaccinated								
1	5·3 (3)	1·6 (1)	1·3 (1)	0	4·1 (3)	1·6 (1)	0	2·0 (9)
3	17·5 (10)	22·6 (14)	25·3 (19)	17·9 (12)	24·3 (18)	11·1 (7)	25·4 (16)	20·8 (96)
4	0	3·2 (2)	1·3 (1)	0	0	6·4 (4)	(0)	1·5 (7)
5	3·5 (2)	4·8 (3)	0	1·5 (1)	0	1·6 (1)	0	1·5 (7)
6A	14·0 (8)	6·5 (4)	12·0 (9)	9·0 (6)	9·5 (7)	17·5 (11)	6·4 (4)	10·6 (49)
6B	1·8 (1)	4·8 (3)	6·7 (5)	1·5 (1)	2·7 (2)	1·6 (1)	3·2 (2)	3·3 (15)
7F	0	0	0	1·5 (1)	0	1·6 (1)	0	0·4 (2)
9V	3·5 (2)	6·5 (4)	1·3 (1)	3·0 (2)	4·1 (3)	4·8 (3)	6·4 (4)	4·1 (19)
14	8·8 (5)	11·3 (7)	6·7 (5)	14·9 (10)	10·8 (8)	14·3 (9)	19·1 (12)	12·2 (56)
18C	1·8 (1)	0	4·0 (3)	3·0 (2)	1·4 (1)	3·2 (2)	3·2 (2)	2·4 (11)
19A	7·0 (4)	12·9 (8)	9·3 (7)	6·0 (4)	6·8 (5)	1·6 (1)	1·6 (1)	6·5 (30)
19F	24·6 (14)	8·1 (5)	21·3 (16)	28·4 (19)	13·5 (10)	17·5 (11)	23·8 (15)	19·5 (90)
23F	12·3 (7)	17·7 (11)	10·7 (8)	13·4 (9)	23·0 (17)	17·5 (11)	11·1 (7)	15·2 (70)
Total	100 (57)	100 (62)	100 (75)	100 (67)	100 (74)	100 (63)	100 (63)	100 (461)

§ There was no recruitment of these age groups during surveys 1-3.

Supplementary tables 7 and 8 show the proportion of total VT carriage attributed to individual vaccine serotypes, among PCV-vaccinated and PCV-unvaccinated children, respectively. Low sample sizes do not allow for a robust analysis of relative change over time. Children 18 weeks to 2 years of age were recruited starting survey 4. The denominator is the total VTs identified in each survey. Surveys 1-7 spanned a period of 3·6 to 7·1 years after Malawi's 12 November 2011 introduction of PCV. For total sample size per survey, refer to Supplementary tables 3 and 4.

Supplementary Table 8. Proportion VT carriage attributed to individual VT (PCV-unvaccinated)

Vaccine serotype	Survey-1 % (n)	Survey-2 % (n)	Survey-3 % (n)	Survey-4 % (n)	Survey-5 % (n)	Survey-6 % (n)	Survey-7 % (n)	Total % (n)
Infants 4-8 weeks, PCV-unvaccinated §								
1	--	--	--	--	0	0	0	0
3	--	--	--	--	7·7 (1)	0	12·5 (1)	6·7 (2)
4	--	--	--	--	0	0	0	0
5	--	--	--	--	0	0	0	0
6A	--	--	--	--	15·4 (2)	25·0 (2)	0	4 (13·3)
6B	--	--	--	--	7·7 (1)	0	37·5 (3)	4 (13·3)
7F	--	--	--	--	0	0	0	0
9V	--	--	--	--	7·7 (1)	0	0	1 (3·3)
14	--	--	--	--	7·7 (1)	25·0 (2)	12·5 (1)	13·3 (4)
18C	--	--	--	--	7·7 (1)	12·5 (1)	0	6·7 (2)
19A	--	--	--	--	0	0	0	0
19F	--	--	--	--	15·4 (2)	25·0 (2)	25·0 (2)	20·0 (6)
23F	--	--	--	--	30·8 (4)	12·5 (1)	12·5 (1)	23·3 (6)
Total	--	--	--	--	100 (13)	100 (8)	100 (8)	100 (29)
Children 3-10 years, PCV-unvaccinated								
1	14·3 (10)	6·0 (3)	2·1 (1)	0	0	5·3 (1)	0	5·6 (15)
3	12·9 (9)	20·0 (10)	23·4 (11)	14·3 (4)	0	26·3 (5)	6·7 (2)	16·1 (41)
4	5·7 (4)	2·0 (1)	10·6 (5)	7·1 (2)	0	15·8 (3)	0	5·9 (15)
5	1·4 (1)	0	0	0	0	10·5 (2)	3·3 (1)	1·6 (4)
6A	10,0 (7)	20·0 (10)	6·4 (3)	21·4 (6)	18·2 (2)	5·3 (1)	13·3 (4)	12·9 (33)
6B	2·9 (2)	10·0 (5)	8·5 (4)	7·1 (2)	9·1 (1)	0	10·0 (3)	6·7 (17)
7F	5·7 (4)	2·0 (1)	2·1 (1)	0	9·1 (1)	5·3 (1)	0	6·7 (8)
9V	8·6 (6)	6·0 (3)	4·3 (2)	7·1 (2)	9·1 (1)	0	6·7 (2)	6·3 (16)
14	0	8·0 (4)	17·0 (8)	7·1 (2)	9·1 (1)	5·3 (1)	10·0 (3)	7·5 (19)
18C	7·1 (5)	4·0 (2)	8·5 (4)	0	27·3 (3)	5·3 (1)	6·7 (2)	6·7 (17)
19A	4·3 (3)	4·0 (2)	4·3 (2)	7·1 (2)	0	0	10·0 (3)	4·7 (12)
19F	12·9 (9)	10·0 (5)	8·5 (4)	10·7 (3)	0	10·5 (2)	16·7 (5)	11·0 (28)
23F	14·3 (10)	8·0 (4)	4·3 (2)	17·9 (5)	18·2 (2)	10·5 (2)	16·7 (5)	11·8 (30)
Total	100 (70)	100 (50)	100 (47)	100 (28)	100 (11)	100 (19)	100 (30)	100 (255)
Adults 18-40 years, HIV-infected on ART, PCV-unvaccinated								
1	3·3 (1)	13·8 (4)	7·7 (3)	0	0	0	0	3·7 (8)
3	23·3 (7)	34·5 (10)	28·2 (11)	27·3 (12)	37·5 (12)	36·0 (9)	55·6 (10)	32·7 (71)
4	13·3 (4)	6·9 (2)	7·7 (3)	2·3 (1)	9·4 (3)	8·0 (2)	0	6·9 (15)
5	0	0	2·6 (1)	0	0	0	0	0·5 (1)
6A	13·3 (4)	3·5 (1)	10·3 (4)	4·6 (2)	6·3 (2)	4·0 (1)	5·6 (1)	6·9 (15)
6B	3·3 (1)	3·5 (1)	0	0	3·1 (1)	4·0 (1)	0	1·8 (4)
7F	0	0	0	4·6 (2)	0	0	0	0·9 (2)
9V	3·3 (1)	3·5 (1)	5·1 (2)	9·1 (4)	3·1 (1)	4·0 (1)	0	4·6 (10)
14	0	0	7·7 (3)	9·1 (4)	3·1 (1)	8·0 (2)	0	4·6 (10)
18C	6·7 (2)	3·5 (1)	0	9·1 (4)	0	8·0 (2)	16·7 (3)	5·5 (12)
19A	10·0 (3)	6·9 (2)	7·7 (3)	9·1 (4)	9·4 (3)	12·0 (3)	5·6 (1)	8·8 (19)
19F	20·0 (6)	17·2 (5)	5·1 (2)	13·6 (6)	15·6 (5)	8·0 (2)	16·7 (3)	13·4 (29)
23F	3·3 (1)	6·9 (2)	18·0 (7)	11·4 (5)	12·5 (4)	8·0 (2)	0	9·7 (21)
Total	100 (30)	100 (29)	100 (39)	100 (44)	100 (32)	100 (25)	100 (18)	100 (217)

§ There was no recruitment of these age groups during surveys 1-4

Supplementary Table 9. Serotype-3: impact of classifying serotype-3 as NVT on carriage prevalence and prevalence ratio

Children 3-5 years (PCV-vaccinated)	Survey 1 (n=286)	Survey 2 (n=303)	Survey 3 (n=361)	Survey 4 (n=378)	Survey 5 (n=371)	Survey 6 (n=382)	Survey 7 (n=324)	Total (n=2405)	cPR¥ (95% CI) p-value	aPR¥ (95% CI) p-value	Relative change
Total carriage<br>% (n)<br>95% CI	84·2 (241)<br>79·5, 88·3	75·9 (230)<br>70·7, 80·6	78·1 (282)<br>73·5, 82·3	67·0 (253)<br>61·9, 71·7	80·6 (299)<br>76·2, 84·5	67·3 (257)<br>62·3, 72·0	79·7 (258)<br>74·8, 83·9	75·7 (1820)<br>73·9, 77·4	--	--	--
VT, Excluding serotype 3	16·4 (47)<br>12·6, 21·2	15·8 (48)<br>12·1, 20·4	15·5 (56)<br>12·1, 19·6	14·6 (55)<br>11·3, 18·5	14·6 (54)<br>11·3, 18·5	13·4 (51)<br>10·3, 17·2	13·0 (42)<br>9·7, 17·1	14·7 (353)<br>13·3, 16·2	0·926 (0·842, 1·018)<br>0·111	0·942 (0·856, 1·036)<br>0·218	-20·7%
NVT, Including serotype-3	67·8 (194)<br>62·2, 73·0	60·1 (182)<br>54·4, 65·4	62·6 (226)<br>57·5, 67·5	52·4 (198)<br>47·3, 57·4	66·0 (245)<br>61·1, 70·7	53·9 (206)<br>48·9, 58·9	66·7 (216)<br>61·3, 71·6	61·0 (1467)<br>59·0, 63·0	0·964 (0·934, 0·996)<br>0·026	0·968 (0·937·1·000)<br>0·048	-1·6%
Children 6-8 years (PCV-unvaccinated)	Survey 1 (n=91)	Survey 2 (n=111)	Survey 3 (n=139)	Survey 4 (n=128)	Survey 5 (n=56)	Survey 6 (n=100)	Survey 7 (n=70)	Total (n=695)	cPR¥ (95% CI) p-value	aPR¥ (95% CI) p-value	
Total carriage<br>% (n)<br>95% CI	67·1 (61)<br>56·4, 76·5	65·8 (73)<br>56·2, 74·5	63·3 (88)<br>54·7, 71·3	43·0 (55)<br>34·3, 52·0	55·3 (31)<br>41·5,.68·7	52·0 (52)<br>41·8, 62·1	61·4 (43)<br>49·0, 72·8	58·0 (403)<br>54·2, 61·7	--	--	--
VT, Excluding serotype 3	22·0 (20)<br>14·6, 31·7	17·1 (19)<br>11·2, 25·3	15·8 (22)<br>10·6, 22·9	12·5 (16)<br>7·8, 19·5	8·9 (5)<br>3·7, 19·9	10·0 (10)<br>5·4, 17·7	15·7 (11)<br>8·9, 26·3	14·8 (103)<br>12·3, 17·7	0·812 (0·680, 0·970)<br>0·021	0·844 (0·699, 1·019)<br>0·078	-28·6%
NVT, Including serotype 3	45·1 (41)<br>35·1, 55·4	48·7 (54)<br>39·4, 57·9	47·5 (66)<br>39·3, 55·8	30·5 (39)<br>23·1, 39·0	46·4 (26)<br>33·8, 59·6	42·0 (42)<br>32·7, 51·9	45·7 (32)<br>34·4, 57·5	43·2 (300)<br>39·4, 46·9	0·948 (0·872, 1·031)<br>0·214	0·959 (0·878, 1·047)<br>0·347	+1·3%
Adult, HIV-infected on ART (PCV-unvaccinated)	Survey 1 (n=198)	Survey 2 (n=201)	Survey 3 (n=279)	Survey 4 (n=308)	Survey 5 (n=305)	Survey 6 (n=277)	Survey 7 (n=202)	Total (n=1770)	cPR¥ (95% CI) p-value	aPR¥ (95% CI) p-value	
Total carriage<br>% (n)<br>95% CI	39·4 (78)<br>32·5, 46·6	47·3 (95)<br>40·2, 54·4	43·4 (124)<br>38·5, 50·5	42·9 (132)<br>37·3, 48·6	38·4 (117)<br>32·9, 44·1	32·5 (90)<br>27·0, 38·4	38·7 (78)<br>31·9, 45·7	40·4 (714)<br>38·0, 42·7	--	--	--
VT, Excluding serotype 3	11·6 (23)<br>7·8-16·9	9·5 (19)<br>6·1-14·4	10·0 (28)<br>7·0-14·2	10·4 (32)<br>7·4-14·3	6·6 (20)<br>4·3-10·0	5·8 (16)<br>3·6-9·2	4·0 (8)<br>2·0-7·7	8·3 (146)<br>7·1-9·6	0·783 (0·673, 0·912)<br>0·002	0·763 (0·655, 0·888)<br>0·000	-65·5%
NVT, Including serotype 3	27·8 (55)<br>22·0, 34·4	37·8 (76)<br>31·4, 44·7	33·4 (96)<br>29·1, 40·2	32·5 (100)<br>27·5, 37·9	31·8 (97)<br>26·8, 37·3	26·7 (74)<br>21·8, 32·2	34·7 (70)<br>28·4, 41·5	32·1 (568)<br>29·9, 34·3	0·970 (0·908, 1·037)<br>0·373	0·966 (0·904, 1·032)<br>0·309	+24·8%

cPR=crude prevalence ratio. aPR=adjusted ratio. CI=confidence interval. VT=vaccine serotype, NVT=non-vaccine serotype. PCV=pneumococcal conjugate vaccine

Supplementary Note 1. Details of the Non-linear regression analysis framework

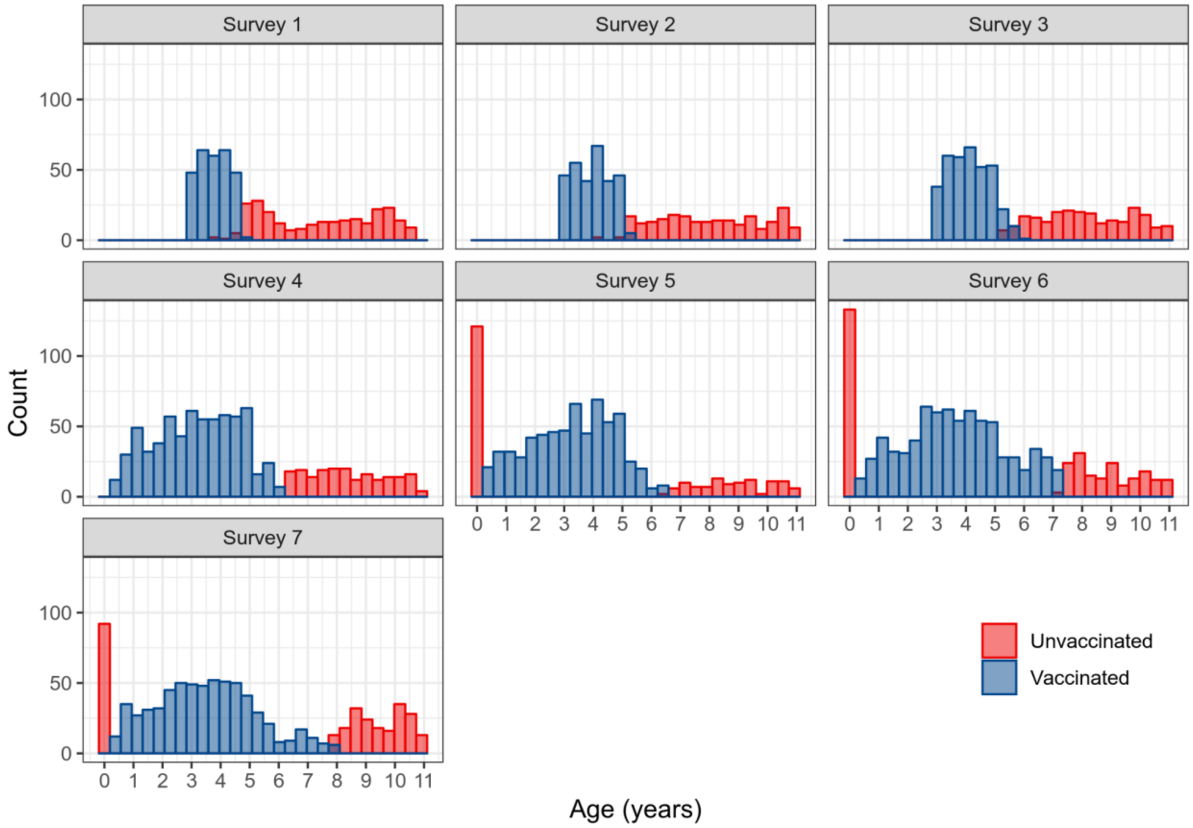

Supplementary Figure 2. Age distribution of PCV-unvaccinated and PCV-vaccinated children. Red and blue bars represent PCV-unvaccinated and PCV-vaccinated children, respectively. The x-axis is age in years old. The y-axis shows the count (frequency) of children. Each bar represents a 4-months progression in age.

Summary description of the non-linear regression analysis: To better understand the rate at which VT and NVT carriage prevalence was decreasing, we developed a model to describe the variation in an individual's probability of VT or NVT carriage with age, adjusted for age at recruitment. The model is fitted using carriage data from children 3·6 to 10 years of age, maximising overlap of empirical data. Model outputs for individual carriage probability were then transformed into a population-level (decay) half-life of each VT and NVT carriage. By calculating the effect of not receiving the vaccination (β), we can then begin to define the benefit of vaccine-induced immunity in lowering an individual's risk of VT carriage at different ages after receiving PCV vaccination. This was then be extrapolated to define the rate of reduced VT carriage within the studied population. Further analyses, not currently available, should include a measure of other contributing factors, including waning maternal and developing naturally acquired immunity.

Model specification, VT

In this cross-sectional study, the response (i.e. VT carrier or not) for each child is a single binary variable, $Y_i = 1/0$ representing presence/absence of VT carriage at the time of measurement (i.e. sample collection), with respective probabilities p_i and $1 - p_i$.

The modelled probability, p_i, of VT carriage for an individual child i is $\alpha\beta\exp\{-\delta_u(age_i - t_c)\}$ if the child is unvaccinated and $\alpha\exp\{-\delta_v(age_i - t_c)\}$ if vaccinated, where age_i is the age in years of child i at time of measurement, t_c is the time at which we censor the data (3.6 years), β is the effect of not receiving the vaccination, δ_u and δ_v are the rate of decay of VT carriage

prevalence with age for unvaccinated and vaccinated children, respectively, and α is the VT carriage prevalence for vaccinated children at time t_c.

All parameters estimated in the model are calculated using the maximum likelihood, as also specified in the main manuscript. Y_i is distributed as a Bernoulli with probability p_i specified according to the equations above. From this we can calculate the likelihood and then find the parameters that maximise that likelihood.

Supplementary Table 10. Summary of parameters

	VT carriage, Yes	VT carriage, No (NVT or no carriage)
Variable for carriage status	1	0
Probability of VT carriage	p_i	$1 - p_i$
**Modelling parameter (p_i) (=probability of VT carriage for an individual child, i)**		
Unvaccinated	$\alpha\beta\exp\{-\delta_u(age_i - t_c)\}$	
Vaccinated	$\alpha\exp\{-\delta_v(age_i - t_c)\}$	

age_i	age in years of child i at time of measurement
t_c	time at which we censor the data (3.6 years)
β	effect of not receiving the vaccination
δ_u	rate of decay of VT carriage prevalence with age for unvaccinated children
δ_v	rate of decay of VT carriage prevalence with age for vaccinated children
α	VT carriage prevalence for vaccinated children at time t_c.

Supplementary Table 11. Alpha and beta values for PCV-vaccinated and -unvaccinated children[1]

Parameter	3·6 years old	4 years old	4·5 years old	5 years old	5·5 years old	6 years old	6·5 years old	7 years old
VT carriage prevalence (with 95% CI) at censoring age (3·6 years) for vaccinated children (α)	0·22<br>0·19, 0·25	0·22<br>0·19, 0·25	0·20<br>0·17, 0·23	0·20<br>0·16, 0·24	0·17<br>0·12, 0·23	0·16<br>0·10, 0·26	0·19<br>0·08, 0·38	0·06<br>0·03, 0·13
VT Carriage prevalence (with 95% CI) at censoring age (3·6 years) for unvaccinated children (α* β)	0·47<br>0·43, 0·51	0·47<br>0·43, 0·51	0·45<br>0·42, 0·48	0·40<br>0·36, 0·45	0·36<br>0·30, 0·41	0·30<br>0·23, 0·40	0·25<br>0·12, 0·45	0·20<br>0·16, 0·27
Effect of not receiving the vaccination (β)	2·21<br>1·51, 2·92	2·21<br>1·51, 2·92	2·26<br>1·57, 2·95	2·0<br>1·38, 2·62	2·13<br>1·29, 2·98	1·85<br>0·83, 2·88	1·33<br>0·23, 2·43	3·39<br>0·59, 6·18

[1]Low sample sizes does not allow for a robust analysis beyond seven years of age. CI= Confidence interval. VT=Vaccine serotype.

Goodness-of-fit and limitations of the non-linear regression analysis

The conventional way to assess goodness-of-fit of a regression model is to plot standardised residuals against predicted (fitted) values; in a well-fitting model, the plot should show no discernible structure. However, individual residuals from a model with binary response are unreliable. Instead, we use grouped residuals as follows. Within each of the unvaccinated and vaccinated sets of children, order the estimated probabilities p_i from smallest to largest. Choose a group size, k. The first grouped predicted value is the sum $s_1 = p_1 + \cdots + p_k$, and the first standardised grouped residual is $r_1 = \{(Y_1 + \cdots + Y_k) - s_1\}/\sqrt{(v_1)}$ where $Y_i = 0/1$ denotes absence/presence of VT carriage in the ith child and $v_1 = p_1(1 - p_1) + \cdots + p_k(1 - p_k)$. The second grouped predicted value and standardised residual are calculated in the same way using the next k ordered p_i, and so on.

Supplementary figure 3 shows the resulting plot, using $k = 125$ and $k = 93$ for unvaccinated and vaccinated children, respectively, to give 15 groups in each of the two sets. The plot indicates a good fit in that (a) there is no discernible relationship between the residual and predicted values, and (b) the range of the residuals, from approximately -2 to +2, is compatible

with their theoretical mean and standard deviation of 0 and 1, respectively, if the model is correct.

The major limitation of the analysis reported here is the inability of our model to extrapolate before the censoring time t_c with a reasonable amount of uncertainty. This is due to the small overlap in the age ranges of vaccinated and unvaccinated children and to the fact that the VT carriage dynamic for vaccinated children is too complex to capture, with the available data, in the early years of life.

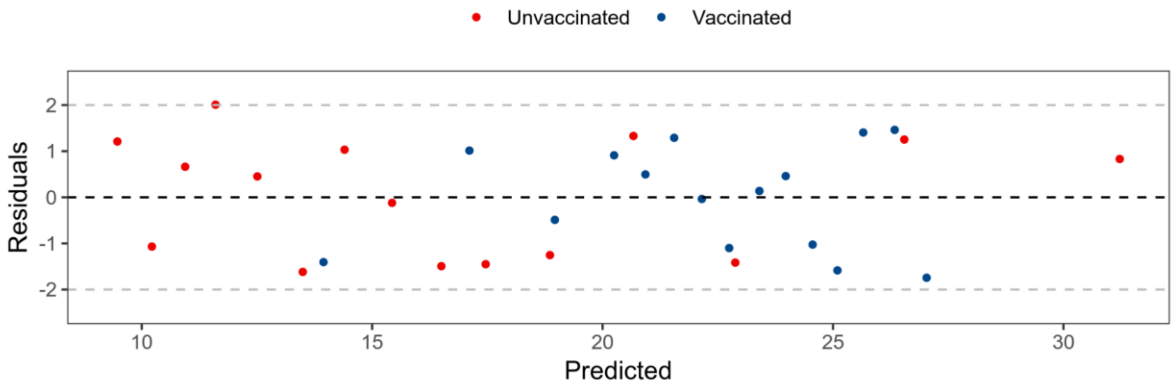

Supplementary Figure 3. Plot of standardised grouped residuals against predicted values. The major limitation of the analysis reported here is the inability of our model to extrapolate before the censoring time t_c with a reasonable amount of uncertainty. This is due to the small overlap in the age ranges of vaccinated and unvaccinated children and to the fact that the VT carriage dynamic for vaccinated children is too complex to capture, with the available data, in the early years of life.

Supplementary References

1 Dunne, E. M. et al. Effect of ten-valent pneumococcal conjugate vaccine introduction on pneumococcal carriage in Fiji: results from four annual cross-sectional carriage surveys. *Lancet Glob. Health* **6**, e1375–1385 (2018).

2 Collins, D. A. et al. Predictors of pneumococcal carriage and the effect of the 13-valent pneumococcal conjugate vaccination in the Western Australian Aboriginal population. *Pneumonia* **9,** 14 (2017).

3 Sigaúque, S. *et al*. Early Declines in Vaccine Type Pneumococcal Carriage in Children Less Than 5 Years Old After Introduction of 10-valent Pneumococcal Conjugate Vaccine in Mozambique. *Pediatr. Infect. Dis. J.* **37,** 1054–1060 (2018).

4 Hammitt, L. L. et al. Effect of ten-valent pneumococcal conjugate vaccine on invasive pneumococcal disease and nasopharyngeal carriage in Kenya: a longitudinal surveillance study. *Lancet* **393,** 2146-2154 (2019).

5 Heinsbroek, E. et al. Pneumococcal carriage in households in Karonga District Malawi, before and after introduction of pneumococcal conjugate vaccination. *Vaccine* **36,** 7369–7376 (2018).

6 Hammitt, L. L. et al. Indirect Effect of Conjugate Vaccine on Adult Carriage of Streptococcus pneumoniae: An Explanation of Trends in Invasive Pneumococcal Disease. *J. Infect. Dis.* **193,** 1487–1494 (2006).

7 Spijkerman, J. *et al.* Carriage of *Streptococcus pneumoniae* 3 Years after Start of Vaccination Program, the Netherlands. *Emerg. Infect. Dis.* **17,** 584-591 (2011).

8 van Hoek, A. J. et al. Pneumococcal carriage in children and adults two years after introduction of the thirteen valent pneumococcal conjugate vaccine in England. *Vaccine* **32,** 4349–4355 (2014).

9 Flasche, S. et al. Effect of Pneumococcal Conjugate Vaccination on Serotype-Specific Carriage and Invasive Disease in England: A Cross-Sectional Study. *PLoS Med.* **8,** e1001017 (2011).
