# Supplementary material for: High residual carriage of vaccine-serotype Streptococcus pneumoniae after introduction of pneumococcal conjugate vaccine in Malawi
Source: Nat Commun. 2020 May 6;11:2222. doi: 10.1038/s41467-020-15786-9 (PMC7203201; doi:10.1038/s41467-020-15786-9)
Supplement: Supplementary file 3 — Reporting Summary [file 41467_2020_15786_MOESM3_ESM.pdf]

nature research

Corresponding author(s): Todd D Swarthout

Last updated by author(s): Mar 16, 2020

Reporting Summary

Nature Research wishes to improve the reproducibility of the work that we publish. This form provides structure for consistency and transparency in reporting. For further information on Nature Research policies, see Authors & Referees and the Editorial Policy Checklist .

Statistics

For all statistical analyses, confirm that the following items are present in the figure legend, table legend, main text, or Methods section.

n/a	Confirmed	
☐	☒	The exact sample size (*n*) for each experimental group/condition, given as a discrete number and unit of measurement
☐	☒	A statement on whether measurements were taken from distinct samples or whether the same sample was measured repeatedly
☐	☒	The statistical test(s) used AND whether they are one- or two-sided<br>*Only common tests should be described solely by name; describe more complex techniques in the Methods section.*
☐	☒	A description of all covariates tested
☐	☒	A description of any assumptions or corrections, such as tests of normality and adjustment for multiple comparisons
☐	☒	A full description of the statistical parameters including central tendency (e.g. means) or other basic estimates (e.g. regression coefficient) AND variation (e.g. standard deviation) or associated estimates of uncertainty (e.g. confidence intervals)
☐	☒	For null hypothesis testing, the test statistic (e.g. *F*, *t*, *r*) with confidence intervals, effect sizes, degrees of freedom and *P* value noted<br>*Give P values as exact values whenever suitable.*
☒	☐	For Bayesian analysis, information on the choice of priors and Markov chain Monte Carlo settings
☒	☐	For hierarchical and complex designs, identification of the appropriate level for tests and full reporting of outcomes
☒	☐	Estimates of effect sizes (e.g. Cohen's *d*, Pearson's *r*), indicating how they were calculated

Our web collection on statistics for biologists contains articles on many of the points above.

Software and code

Policy information about availability of computer code

Data collection	Participant data collection was completed using Open Data Kit (ODK) Collect open source software. (v1.24.0).
Data analysis	Statistical analyses were completed using Stata 13.1 (StataCorp, College Station, TX, USA). Non-linear regression analysis was completed using R open-source software, version 3.5.0.

For manuscripts utilizing custom algorithms or software that are central to the research but not yet described in published literature, software must be made available to editors/reviewers. We strongly encourage code deposition in a community repository (e.g. GitHub). See the Nature Research guidelines for submitting code & software for further information.

Data

Policy information about availability of data

All manuscripts must include a data availability statement. This statement should provide the following information, where applicable:

- Accession codes, unique identifiers, or web links for publicly available datasets
- A list of figures that have associated raw data
- A description of any restrictions on data availability

The data supporting the findings of this study has been deposited in the Figshare repository (DOI: 10.6084/m9.figshare.11985255) (ref.72). The source data underlying Figures 2 and 3 are available in the Supplementary Information, Supplementary Tables 3 and 4, respectively. Fig.4 data is available with the coding data at: https://github.com/claudiofronterre/pneumococco

Field-specific reporting

Please select the one below that is the best fit for your research. If you are not sure, read the appropriate sections before making your selection.

Life sciences study design

All studies must disclose on these points even when the disclosure is negative.

Sample size	The sample size strategy was pragmatic to allow for adequate precision of the carriage prevalence estimates. VT carriage was considered the primary endpoint, and the sample size was calculated based on the precision of the prevalence estimation, assuming an infinite sampling population. Among children 3–7 years old (vaccinated), an absolute VT prevalence up to 10% was expected, with a sample of 300/survey providing a 95% confidence interval (CI) of 6·6%–13·4%. Among children 3–10 years old (unvaccinated) and HIV-infected adults, an absolute VT prevalence of 20% was expected, with a sample of 200/survey providing a 95% CI of 14·5%–25·5%.
Data exclusions	No data were excluded.
Replication	There were no formal experimental findings in this observational study.
Randomization	This was an observational, non-interventional, study. Infants 4-8 weeks were recruited from vaccination centres using systematic sampling. Children 18 weeks–7 years were recruited from households within pre-defined clusters. Both cluster and household were randomly selected. Within each cluster, after randomly choosing a first house using a 'spin the bottle' technique, teams moved systematically, recruiting one eligible child per household until the required number of children were recruited from each cluster. Children 5-10 years old were recruited from schools. A computer-generated randomized number list was used to select children from the school register. HIV-infected adults 18–40 years old and on ART, were recruited from Blantyre's Queen Elizabeth Central Hospital ART Clinic using systematic sampling.
Blinding	In this observational study, the only blinding that occurred was in blinding the laboratory technicians to the source of collected samples.

Reporting for specific materials, systems and methods

We require information from authors about some types of materials, experimental systems and methods used in many studies. Here, indicate whether each material, system or method listed is relevant to your study. If you are not sure if a list item applies to your research, read the appropriate section before selecting a response.

Materials & experimental systems

n/a	Involved in the study	
☒	☐	Antibodies
☒	☐	Eukaryotic cell lines
☒	☐	Palaeontology
☒	☐	Animals and other organisms
☐	☒	Human research participants
☒	☐	Clinical data

Methods

n/a	Involved in the study	
☒	☐	ChIP-seq
☒	☐	Flow cytometry
☒	☐	MRI-based neuroimaging

Human research participants

Policy information about studies involving human research participants

Population characteristics	This observational filed-based study occurred in medium- and high-density townships of urban Blantyre, Malawi. We recruited: i) healthy (reported) children aged 4 weeks to 10 years, regardless of gender (50.3& male [2708/5378] & 49.7% female (2670/5378) and ii) HIV-infected adults 18-40 years old and on antiretrovial therapy (ART), regardless of gender (68% male [1141/1770] & 32% female [559/1770]).
Recruitment	Participation was voluntary, though participation of screened individuals was high. For children recruited from schools, letters were sent home inviting parents/guardians to travel to the school to discuss the study and consider consenting to their child's participation. If the parent/guardian did not respond to the letter or did not visit the school within the specified 3 days, another child was randomly selected from the school's register. The number of letters actually received by the parents/guardians and the reasons for parents/guardians not accepting the invitations were not routinely documented. During household recruitment, study teams maintained a diary to record the number of homes visited. An average of 7·2 households were approached for every child screened. HIV-infected adults 18–40 years old and on ART were recruited from Blantyre's Queen Elizabeth Central Hospital ART Clinic using systematic sampling. Though difficult to determine, it is not expected that any self-selection would significantly influence the study findings.
Ethics oversight	The study protocol was approved by the College of Medicine Research and Ethics Committee, University of Malawi (P.02/15/1677) and the Liverpool School of Tropical Medicine Research Ethics Committee (14.056).

Note that full information on the approval of the study protocol must also be provided in the manuscript.
